# Supplementary material for: Mo2C-Loaded Porous Carbon Nanosheets as a Multifunctional Separator Coating for High-Performance Lithium–Sulfur Batteries
Source: Materials (Basel). 2023 Feb 15;16(4):1635. doi: 10.3390/ma16041635 (PMC9964068; doi:10.3390/ma16041635)
Supplement: Supplementary file 1 [file materials-16-01635-s001.zip › materials-2172673-supplementary.pdf]

Supporting Information

Mo_2C-Loaded Porous Carbon Nanosheets as a Multifunctional Separator Coating for High-Performance Lithium–Sulfur Batteries

Jianli Zhang, Yang Wang, Zhenkai Zhou, Qiang Chen and Yiping Tang *

College of Material Science and Engineering, Zhejiang University of Technology, Hangzhou 310014, China
* Correspondence: tangyiping@zjut.edu.cn

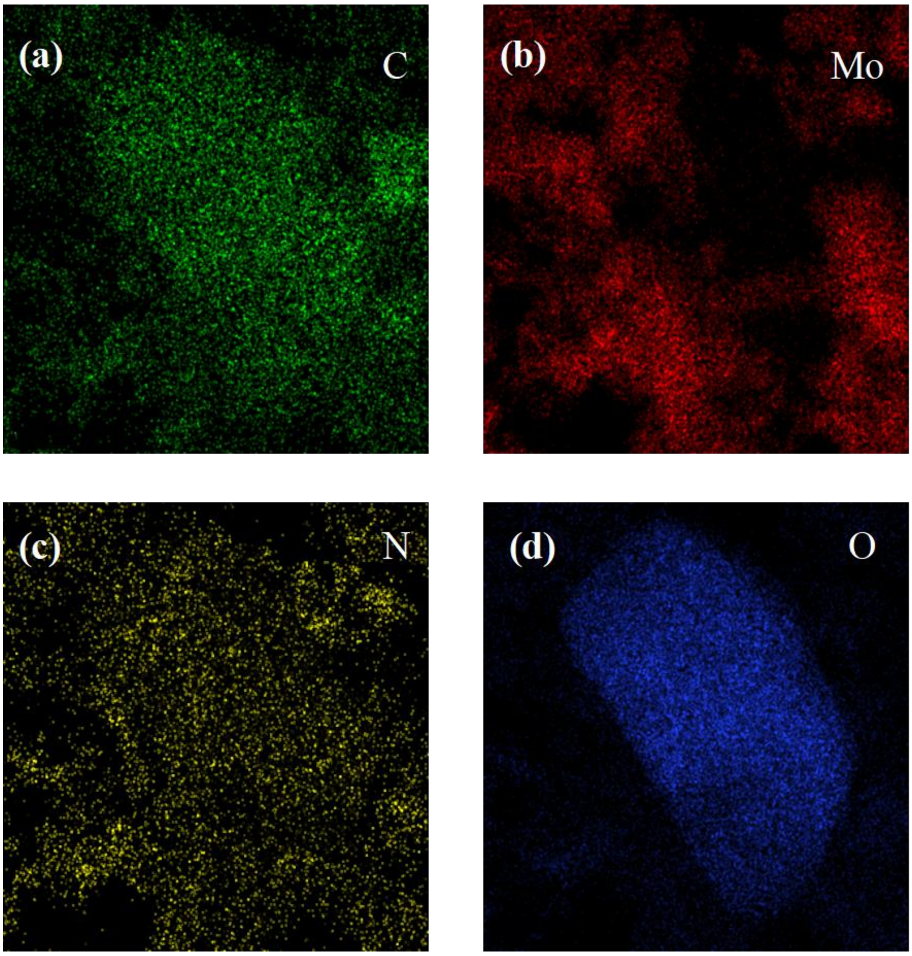

Figure S1. TEM-EDX elemental mapping of Mo_2C/C (a) C, (b) Mo, (c) N, and (d) O.

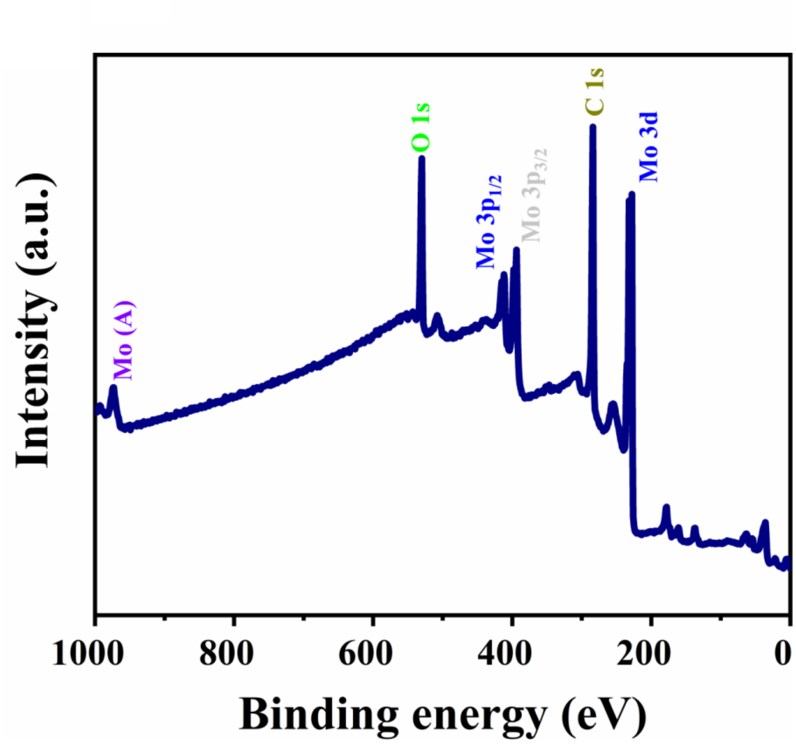

Figure S2. XPS spectra of the Mo_2C/C full spectrum.

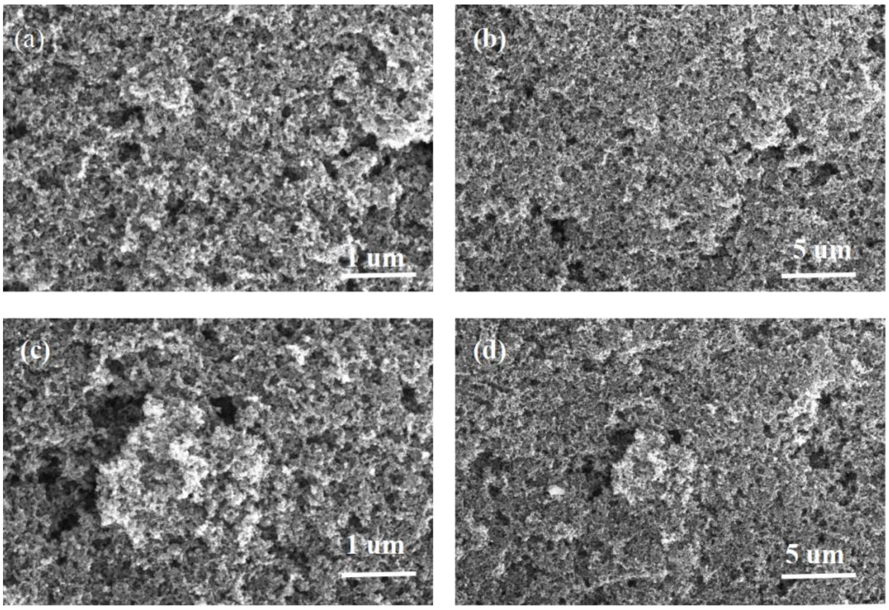

Figure S3. SEM images of carbon separator: (a-b) before cycling, (c-d) after the 100th cycle.

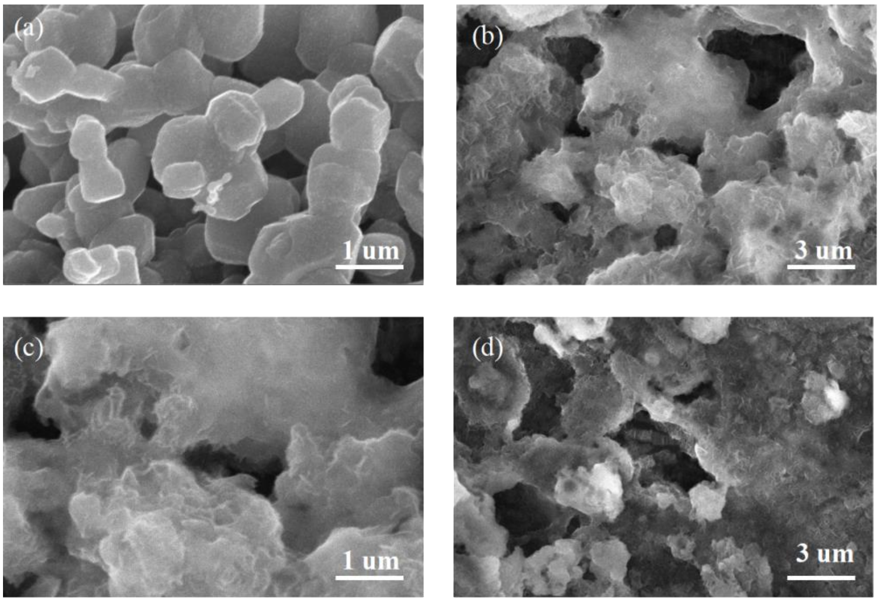

Figure S4. SEM images of Mo2C-C separator: (a-b) before cycling, (c-d) after the 100th cycle.

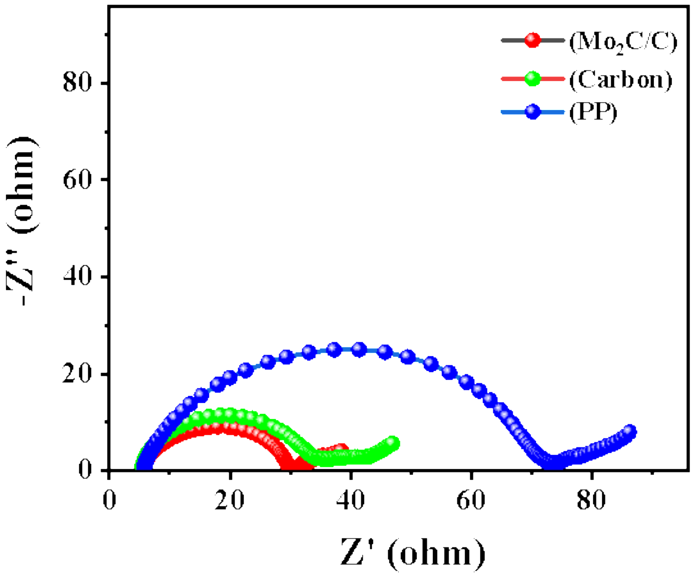

Figure S5. EIS spectra of different separators.

Table S1. Electrochemical performances of this work compared with previous works involving different separators in recently reported literature.

Modified separator	*S loading (mg cm^{-2})*	*Coating thickness (μm)*	*Coating loading (mg cm^{-2})*	*Rate capacity (mAh g^{-1})*	*Capacity decay rate/cycle number/ C rate*	*Ref.*
N,S-Mo2C/C-ACF //PP	1	6	0.5	900 (1C)	0.08%/600/1C	[1]
KB/Mo2C //PP	1.2	25	0.6	664 (1C)	0.076%/ 600/1C	[2]
Ni-Co-P@C//PP	1.8	3.2	0.4	906 (1C)	0.056%/1000/0.5C	[3]
FeP//PP	1.9-2.0	15.6	1.3	791.1 (1C)	0.038%/1500/1C	[4]
MnO_2/CNT//PP	0.8	10	0.35	794.2 (1C)	0.136%/500/1C	[5]
VN//PP	1.6	25	1.52	760 (2C)	0.077%/800/1C	[6]
CuS/G//PP	1.85	19.5	0.5	568 (3C)	0.19%/200/1C	[7]
MWCNTs/NCQDs//PP	1.3-1.5	-	0.015	895 (1C)	0.056%/1000/1C	[8]
Mo_2C/C//PP	1-1.4	20	0.5	930 (1C)	0.09%/400/1C	This work

References

1. Li, H.; Jin, Q.; Li, D.; Huan, X.; Liu, Y.; Feng, G.; Zhao, J.; Yang, W.; Wu, Z.; Zhong, B.; Guo, X.; Wang, B. Mo_2C embedded carambola-like N,S-rich carbon framework as interlayer material for high-rate lithium-sulfur batteries in wide temperature range. ACS Appl. Mater. Inter. **2020**, *20*, 22971–22980.
2. He, M.; Li, X.; Li, W.; Zheng, M.; Wang, J.; Ma, S.; Ma, Y.; Yin, G.; Zuo, P.; Sun, X. Immobilization and kinetic promotion of polysulfides by molybdenum carbide in lithium-sulfur batteries. Chem. Eng. J. **2021**, *411*, 128563.
3. Wu, Z.; Chen, S.; Wang, L.; Deng, Q.; Zeng, Z.; Wang, J.; Deng, S., Implanting nickel and cobalt phosphide into well-defined carbon nanocages: A synergistic adsorption-electrocatalysis separator mediator for durable high-power Li-S batteries. Energy Storage Mater. **2021**, *38*, 381-388.
4. Ma, M.; Cao, L.; Yao, K.; Li, J.; Kajiyoshi, K.; Huang, J. Tailoring FeP with a hollow urchin architecture for high-performance Li-S batteries. ACS Sustain. Chem. Eng. 2021, 9, 5315-5321.
5. Wang, Y.; Liu, W.; Liu, R.; Pan, P.; Suo, L.; Chen, J.; Feng, X.; Wang, X.; Ma, Y.; Huang, W. Inhibiting polysulfide shuttling using dual-functional nanowire/nanotube modified layers for highly stable lithium-sulfur batteries, New J. Chem. **2019**, *43*, 14708-14713.
6. Song, Y.; Zhao, S.; Chen, Y.; Cai, J.; Li, J.; Yang, Q.; Sun, J.; Liu, Z. Enhanced sulfur redox and polysulfide regulation via porous VN-modified separator for Li-S batteries, ACS Appl. Mater. Inter. **2019**, *11*, 5687-5694.
7. Li, H.; Sun, L.; Zhao, Y.; Tan, T.; Zhang, Y. A novel CuS/graphene-coated separator for suppressing the shuttle effect of lithium/sulfur batteries, Appl. Surf. Sci. **2019**, *466*, 309-319.
8. Pang, Y.; Wei, J.; Wang, Y.; Xia, Y., Synergetic protective effect of the ultralight MWCNTs/NCQDs modified separator for highly stable lithium-sulfur batteries. Adv. Energy Mater. **2018**, *8*, 1702288.
